# What is so hard about abstract words, anyways? A neuromechanistic explanation using brain-constrained neural network models

**DOI:** 10.1007/s11571-026-10542-z

**Published:** 2026-09-24

**Authors:** Fynn R. Dobler, Lorenzo Stroppa, Friedemann Pulvermüller

**Affiliations:** 1 Brain Language Laboratory, Department of Philosophy and Humanities, WE4, Freie Universität Berlin, Habelschwerdter Allee 45, 14195 Berlin, Germany; 2 Einstein Center for Neurosciences, Charité – Universitätsmedizin Berlin, 10117 Berlin, Germany; 3 Berlin School of Mind and Brain, Humboldt Universität zu Berlin, 10099 Berlin, Germany; 4 Cluster of Excellence ‘Matters of Activity. Image Space Material’, Humboldt Universität zu Berlin, 10099 Berlin, Germany

**Keywords:** Concept acquisition, Abstract words, Abstract concepts, Spiking neural network, Hebbian learning

## Abstract

It is well known that children first learn words with concrete meaning, whereas abstract ones are typically acquired later. To obtain clues about the underlying causes and mechanisms, a brain-constrained neural network model of brain regions involved in semantic processing was trained on instances of concrete and abstract concepts. Model areas contained excitatory and inhibitory neurons, with Hebbian and anti-Hebbian synaptic plasticity rules. This allowed for the formation of neuronal circuits representing different concepts. Concept formation was simulated based on the similarity structure of objects, actions and scenes exemplifying instances of the concept. Subsequently, conceptual instances were co-presented with wordforms to establish semantic links and direct grounding of symbols in the world. Instances of concrete concepts (e.g. different DOGs or HAMMERs) lead to conceptual representations in the form of reverberant neuronal circuits. Such concept formation was impossible for abstract concepts (e.g. BEAUTY, FORCE): the resultant neuronal circuits failed to reverberate and only responded selectively to their grounding instances. When learning wordforms for the different concepts, abstract words developed fully functional neural circuits, comparable with those of concrete words. However, the formation of these circuits was delayed. While concrete concepts were learned preverbally and could then be ‘labelled’ by a word, abstract concepts only emerged when learned with a wordform. This suggests that the delay in abstract word acquisition might be caused by structural dissimilarities between the real-world events these words are used to speak about.

**Supplementary Information:**

The online version contains supplementary material available at https://doi.org/10.1007/s11571-026-10542-z.

## Introduction

Early language acquisition is a marvellous feat: within the first 12 to 18 months, infants rapidly develop the ability to communicate basic needs, name a variety of objects and produce their first simple sentences. This development does not happen in isolation: at the same time that an infant learns to name an ‘apple’, they must also learn what an apple *is*. In fact, throughout all of first language acquisition, the learner must acquire the concept, a word for it and the correct association of concept to word. This extends past rather concrete concepts and terms – such as *apple*, *hammer* or *dog* – to more abstract ones, such as *democracy*, *beauty* or *cause*. Many researchers agree that abstract words are acquired later than concrete ones (Bellagamba et al. 2022; Bergelson and Swingley 2012, 2013; Brown 1957; Hopkins & Schwanenflugel, 1993; Ponari et al. 2020; Schwanenflugel 1993). But how much later, and why?

**Fig. 1:**
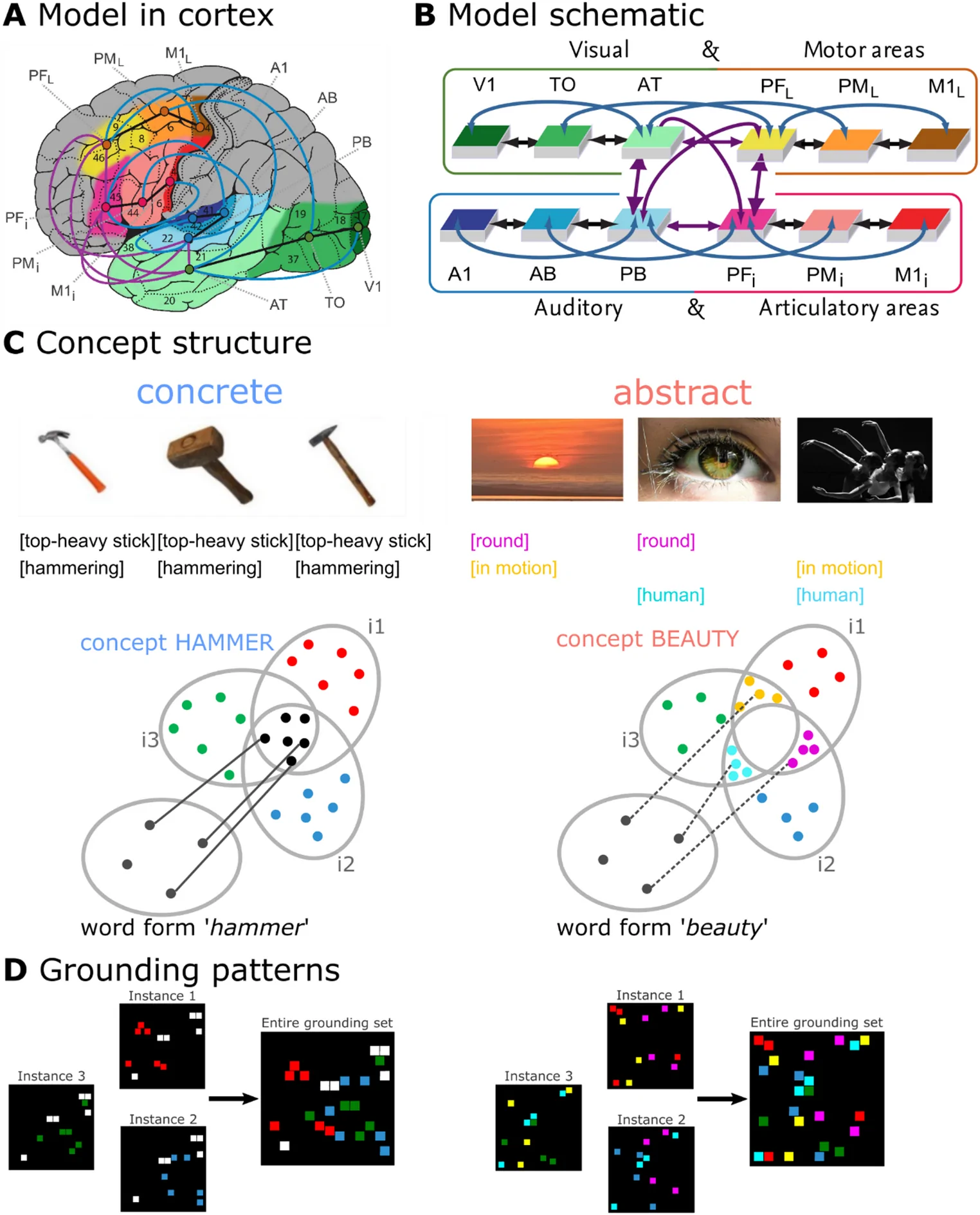
**A** Structure and connectivity of the neural network model. In total, 12 brain areas were modeled, including areas in frontal, temporal, and occipital cortex. Perisylvian areas comprise an inferior frontal articulatory system (red colors) and a superior temporal auditory system (blue colors), and extrasylvian areas comprise a lateral dorsal hand motor system (yellow/brown) and a visual “what” stream of object processing (green). Numbers refer to Brodmann areas, and the colored lines represent long distance cortico-cortical connections as documented by neuroanatomical studies. **B** Schematic depiction of the brain areas modeled (using the same coloring for different brain areas as in (**A**)), along with their connectivity structure. Arrows indicate between-area connections. **C** Top: Examples of overlapping experiential features of concrete (left) and abstract (right) concepts. Bottom: Schematic illustration of the structural difference (full vs. pairwise semantic feature overlap). **D** Specific activity patterns used in this study. Colors indicate whether a simulated neuron is part of one (blue, red, green), two (yellow, cyan, magenta) or all three instances (white)

**Fig. 2:**
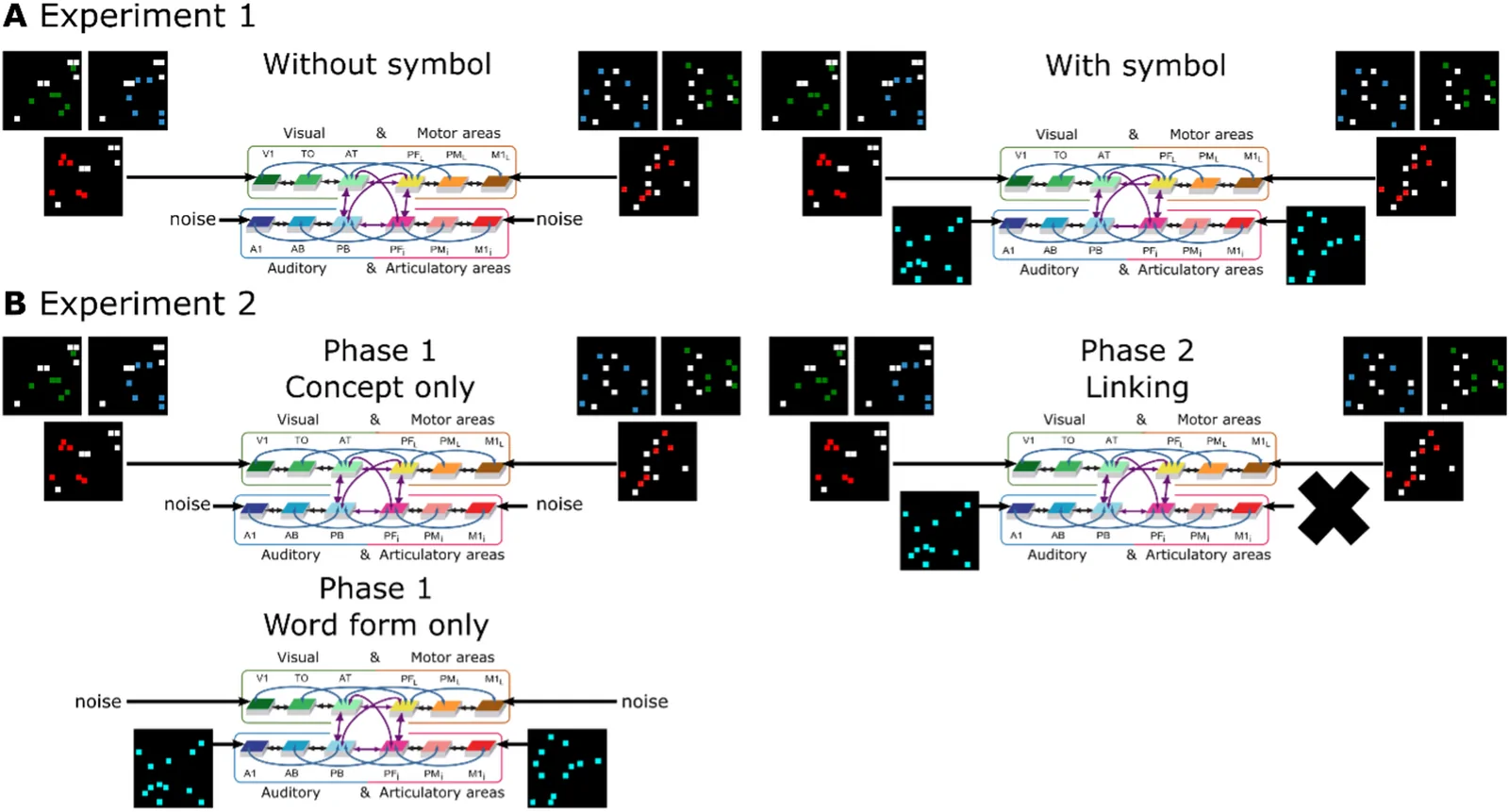
Training regimes for both experiments. Colored dots indicate whether neural elements are related to instance-specific (blue/green/red), semantic/conceptual (white), or wordform (cyan) information. For each concept, three instance-related grounding patterns were presented separately to the network in random order. **A** Experiment 1: Simultaneous concept and wordform acquisition. In the “concept learning” condition (left), only instance activation patterns were presented. In the “semantic learning” condition (right), a wordform pattern was presented together with one of the instance-related patterns of the related concept. **B** Experiment 2: Two-phase concept and wordform acquisition. In Phase 1 (left), instance activation patterns and wordform activation patterns were presented in separate trials. In Phase 2 (right), instance activation patterns were presented simultaneously with the spoken wordform pattern

**Fig. 3:**
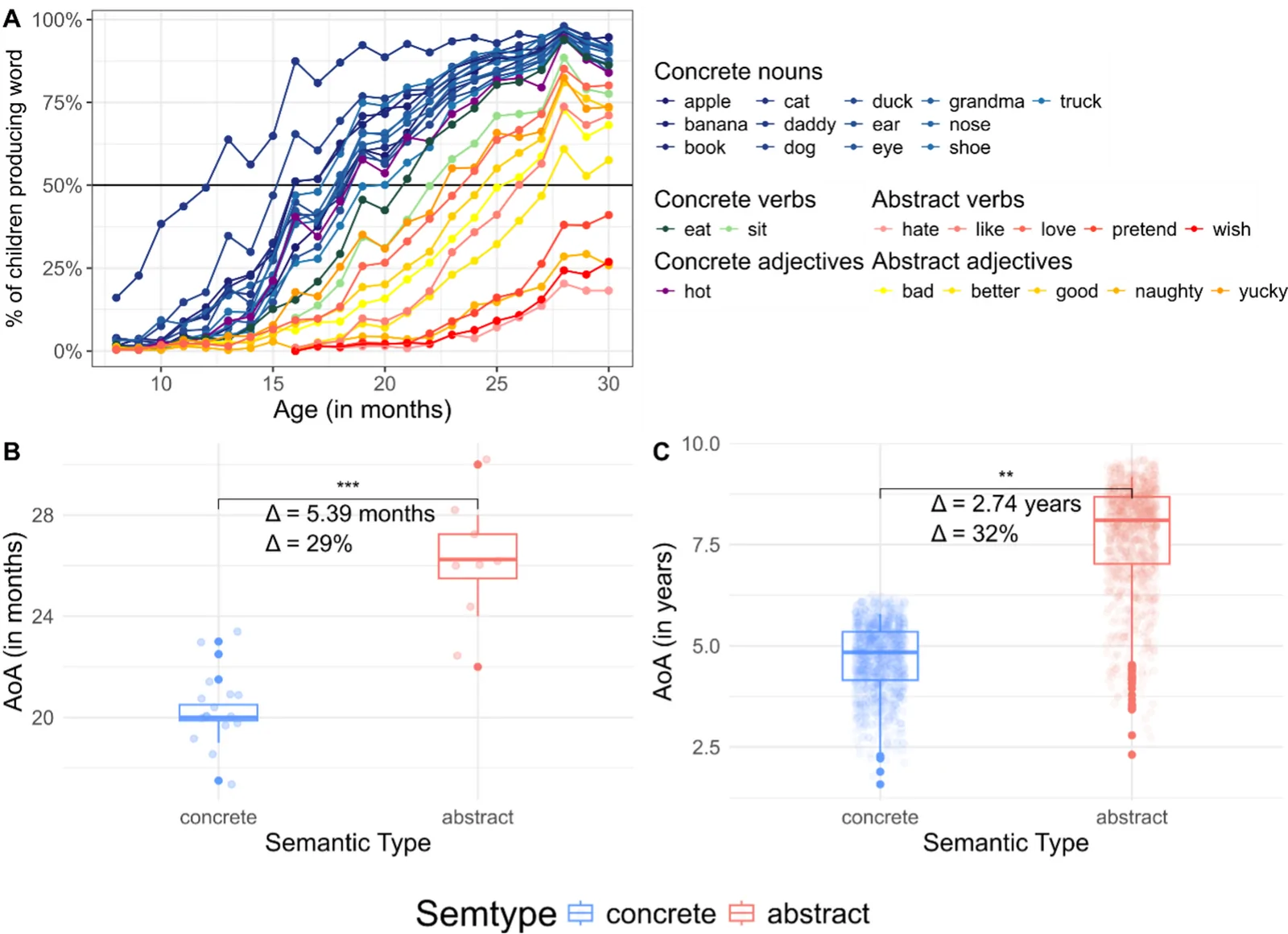
**A** Production trajectories for the concrete and abstract words with the lowest AoA in Wordbank. While all abstract words in the questionnaire have been selected, selection of concrete words was proportional to their share in the dataset. **B** Estimated AoA for concrete and abstract words shown in Fig. 3A. **C** Estimated AoA for all concrete and abstract words in Kuperman et al. (2012)

Theorizing about underlying causes requires a good understanding of the actual delay, but detailed discussions of the age-of-acquisition (AoA) of concrete vs. abstract terms face a serious challenge: different estimates of word AoA have been obtained with highly variable methods, to the extent that generalization remains difficult. Current datasets can be classified as either retrospective or simultaneous with regards to early language acquisition. Retrospective data comes from rating studies conducted with adult speakers reporting on their autobiographical memory (i.e. Kuperman et al. 2012), while simultaneous data comes from either parent-reported assessments of their child’s lexicon (i.e. Frank et al. 2017) or behavioural studies on toddlers and children throughout language acquisition (i.e. Bergelson and Swingley 2012) (Figs. 1, 2 and 3).

Rating studies with adult speakers are crucial sources of AoA estimates for words that are acquired relatively late, but their resolution during early stages of language development within the first 24 months is lacking. In Kuperman et al. (2012), only two words are reported as acquired before 2 years of age: *mama* and *momma*. This self-reported AoA is in direct opposition to observations from both simultaneous behavioural studies and from parent-reported child lexicon assessments, with the latter showing a productive vocabulary of approximately 300 words for the 50% quantile of 24-month-olds (Frank et al. 2017). While data collected simultaneously to language acquisition has a higher credibility for early AoA estimates, the number of abstract words in the most prominent child lexicon assessment, the MacArthur-Bates Communicative Development Inventories (MB-CDI) (Fenson et al. 1993), is limited. In fact, not a single abstract noun is queried by the MB-CDI, and only a small number of abstract predicates (*like*, *love*, *good*, *pretend*, …). Estimating how much later abstract words are acquired is thus currently not possible with high confidence. However, one can compare the AoA differences that retrospective and simultaneous methods report. Are abstract words in the first two years of life similarly delayed as the abstract words reported in Kuperman et al. (2012)?

Why would concrete words be acquired prior to abstract ones? What differences are there between these stimuli? For this question, we have identified three focal points of discussion: (1) linguistic properties of concrete and abstract *words*, (2) communicative utility of concrete and abstract words throughout language learning, and (3) differences between the concrete and abstract *concepts* connected to these words. We will briefly discuss each of these differences.

**Fig. 4:**
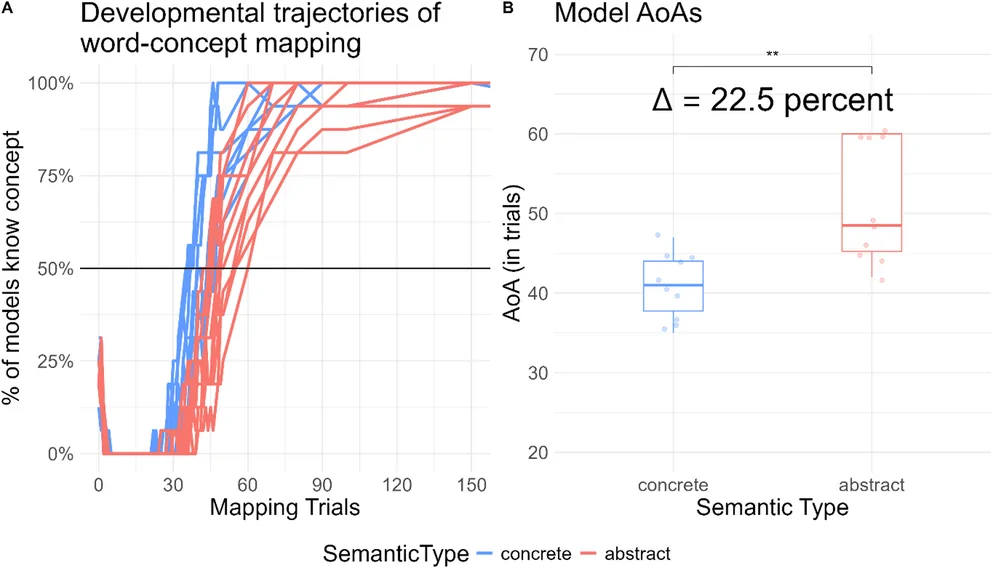
**A** Developmental trajectories of word-concept mapping. In line with trajectories reported in AoA literature, we plot a population-wide acquisition average against ‘model age’ (number of mapping trials). Each line corresponds to a single concrete (blue) or abstract (red) concept. We consider a word mapped to its concept within a model if auditory stimulation elicits activity in > = 30% of the extrasylvian concept cell assembly. For the AoA estimation, we consider it acquired at the trial when > = 50% of all models have successfully mapped concept and word. **B** AoA (in trials) for concrete and abstract concepts. Note that the AoA estimate considers a cluster of abstract concepts acquired at trial 60. Most of these are likely acquired between trial 50 and 60, but due to the models only being tested every 10 training trials after trial 50, there is no reliable estimate of the real AoA

In general, several linguistic properties are correlated to both low AoA and concreteness. When comparing concrete and abstract *words*, concrete words are more frequent, shorter, and have fewer syllables (Lewis and Frank 2016). Shorter, more frequent words are likely easier to learn. However, frequency is a circular predictor for AoA: if a word is acquired relatively early, it will have been experienced and used more often than a word with high AoA (Schwanenflugel 1993). This might in turn affect the measured word frequency. Additionally, word length is not an immediately convincing factor, as early MB-CDI data shows notable differences in the acquisition trajectory of short words with equal length (i.e. *hot* vs. *bad*, see Fig. 4A).

Trivially, concrete words are more useful than abstract ones during early language acquisition. Communicating concrete needs is vastly more necessary for parent-infant communication than discussing abstract ideas. Nonetheless, later acquisition data from Kuperman et al. (2012) shows that even later in life, once basic communicative needs are met, abstract words are still acquired with a similar relative delay (see Fig. 4B).

With both linguistic and communicative utility being insufficient for explaining the difficulty of acquiring abstract words throughout a lifetime of learning, a final possible difference between concrete and abstract words lies in their semantic content. Recent reviews (Borghi et al. 2022; Reilly et al. 2024) summarize that, compared to concrete words, abstract words are viewed as relatively less grounded in action and perception, more related to inner states and social interaction, more reliant on language and communication, and that their meaning is more variable across texts, individuals and throughout an individual’s lifetime (Muraki et al. 2023; Reggin et al. 2021).

The high variability in meaning and the high reliance on context could stem from what some authors describe as structural differences between concrete and abstract concepts (Langland-Hassan et al. 2021; Lupyan and Mirman 2013; Sloutsky 2010): individual instances of concrete concepts, such as different *hammers*, share more object- and action-related semantic features than instances of abstract concepts, such as different things that might be described as *beautiful*. Some authors argue that instances of abstract concepts lack shared grounded features entirely (Buccino et al. 2019; Löhr 2022). However, while there is some population-wide disagreement as to what qualifies as BEAUTIFUL, people are generally able to classify examples as BEAUTIFUL with similar ease as classifying something as a HAMMER. Clearly, there are some verifiable external properties to even abstract concepts. But what could those be?

Consider a selection of things that might be BEAUTIFUL: the eye of a loved one, a sunset, and a dance performance. There does not appear to be a single criterial feature that identifies these three things as BEAUTIFUL. However, there are some properties that subsets of ‘beautiful things’ share. For example, both the eye and the sunset prominently feature something ROUND, while the dance performance does not. Both the dance performance and the eye prominently feature another HUMAN, while the sunset does not, and both the sunset and the dance performance are events with a necessary MOTION component, while the eye is not (see Fig. 1C for an illustration; see also Pulvermüller 2013; Pulvermüller 2018 and Pulvermüller 2023). With the scope of abstract concepts usually being larger than that of concrete ones, these partially shared subsets of features can likely be found in most abstract concepts, as observed by Wittgenstein (1953). He used the example of GAME: while different games do not appear to share a single criterial feature, there are some shared commonalities between types of game. Some games require to be played with other people (e.g. soccer, poker, theater plays), while others are single-player only (e.g. solitaire, Tetris). Some games require elements of randomization (e.g. poker, Tetris, most dice games), others do not (e.g. chess, tic-tac-toe, go), and some other games require physical ability to play well (e.g. soccer, Tetris, sports in general). While these instances of GAME clearly have some shared commonalities, they are not present in all instances.

As a child learns to use words correctly, this difference in semantic structure might facilitate the acquisition of concrete meanings and/or delay the acquisition of abstract ones. Notably, these differences in semantic structure can be formulated in terms of feature correlation (Pulvermüller 2018). If concrete concepts are characterized by instances with high within-concept feature correlation, their acquisition and association with words might be able to exploit associative learning mechanisms better than abstract concepts. Associative learning is supported by synaptic plasticity rules like long-term potentiation (LTP) and long-term depression (LTD) (Artola and Singer 1993; Hebb 1949; Song et al. 2000; Stacho and Manahan-Vaughan 2022). Is the difference in feature correlation between concrete and abstract concepts sufficient to explain the delay in abstract word learning, or must we assume more complex mechanisms leading to the delay?

Investigating the role of semantic feature correlation and associative learning independently from other confounding factors in vivo faces significant methodological challenges: first, separating semantic structures from linguistic properties of concrete and abstract *words*, as well as their communicative utility throughout language acquisition, is notoriously challenging. Furthermore, associative learning is likely affected by the co-occurrence of the target word and a salient potential referent for the infant (Bergelson and Swingley 2013). An ideal experiment would thus control linguistic and pragmatic properties, word-referent co-occurrence rates and sensorimotor features of instances of the target concepts. However, even with all these confounds accounted for, it would be crucial to test *concept* and *word* knowledge separately. While concept knowledge for concrete concepts is frequently probed through visual stimuli, non-verbal tests for abstract concept knowledge are sparse and not easily applicable to infants and children first acquiring language (Fedorenko et al. 2024; Langland-Hassan et al. 2021).

A first step to answer the question posed above is to test the feasibility of LTP and LTD-driven learning for word and concept acquisition through computational modelling. Computational modelling allows for a highly controlled learning experiment and provides an insight into putative neural mechanisms that underly this cognitive phenomenon. To isolate the semantic structure from any confounding factors such as word frequency, word length or the rate of co-occurrence of a word and an instance of its meaning in naturalistic learning situations, we implemented a concept and semantic learning task in a brain-constrained neural network (BCNN). These neural networks are designed according to established neurobiological and -physiological properties of the human brain at the micro-, meso- and macroscopic scale, which gives them higher biological plausibility than networks without these constraints (Pulvermüller et al. 2021). Previous research using BCNNs of semantic learning has shown that their simulated dynamics are in line with semantic learning outcomes in humans by reproducing spatiotemporal differences in the neural representation of object and action words (Tomasello et al. 2017, 2018, 2019), rapidly learning novel word-referent mappings(Constant et al. 2023) and highlighting differences in conceptual processing induced by using category terms or proper names (Nguyen et al. 2024). In addition to tight control over confounds, BCNNs also allow for (a) acquiring concepts and words separately through different learning paradigms and (b) assessing concept and word knowledge separately by stimulating either auditory or visual or motor areas of the model, and by assessing the response profiles of individual artificial neurons.

Recent work has investigated the properties of neural representations of concrete and abstract concepts in BCNNs (Dobler et al. 2024; Henningsen-Schomers et al. 2023; Henningsen-Schomers and Pulvermüller 2022). The neural circuits representing concrete concepts tended to be large cell assemblies (CAs) that respond to any instance of the concept, whereas abstract concepts tended to be represented by neural circuits that only respond to a subset of concept instances (Henningsen-Schomers and Pulvermüller 2022). Additionally, the neural circuits of concrete concepts tended to remain active for a long time after ignition, reverberating due to the strong synaptic connections between concept-wide neurons, whereas the activity of neurons responding to instances of abstract concepts ceases rapidly (Dobler et al. 2024). These results indicate that BCNNs cannot form functional conceptual representations for abstract concepts without words but can do so for concrete concepts. This outcome is in line with research on prelinguistic infants and animals, which shows that some concepts may be learned through experiential grounding independently of language learning (Mandler 2004; Pearce 2008; Perszyk and Waxman 2018; Pusch et al. 2023).

In BCNNs, these structural and functional differences between concrete and abstract concepts representations disappear when concepts are learned simultaneously with concept terms (Dobler et al. 2024; Henningsen-Schomers et al. 2023). However, it is unclear whether the acquisition trajectories of concrete and abstract words differ in BCNNs. When do concrete concept CAs begin to differ from abstract ones, and when do differences disappear for concrete and abstract word CAs? If trajectories indeed differ in BCNNs, it would be an indication that differences in feature correlation – even if all other confounds are the same – are sufficient to lead to acquisition delays.

In this paper, we investigate the following questions: (1) Are the relative differences in concrete and abstract word acquisition comparable early and later in life? (2) Do BCNNs, trained on concrete and abstract concepts and words that only differ in their semantic feature structure, show a delay for abstract word acquisition? (3) If yes, what neuronal mechanisms cause the delay? (4) If yes, is the delay similar to the one found in empirical human data?

## Methods

### AoA estimation

To estimate the AoA difference of concrete and abstract words, we rely on several publicly available datasets containing empirical AoA estimates (Frank et al. 2017), AoA ratings (Kuperman et al. 2012) and concreteness ratings (Brysbaert et al. 2013) derived from human participants. The data collection in these studies was carried out in accordance with relevant ethical guidelines.

To quantify the AoA difference, we compared early AoA estimates within the first 30 months from the parent-questionnaire based Wordbank dataset (Frank et al. 2017), and AoA ratings from an adult population (Kuperman et al. 2012). This allows us to identify whether parent-reported and self-rated vocabulary acquisition lead to consistent conclusions about any differences between concrete and abstract words. Because the databanks do not distinguish between concrete and abstract words, we operationalized concreteness/abstractness as the top and bottom quartile of the Brysbaert et al. (2013) concreteness ratings (concrete: rating > = 4.08, abstract: <=2.25).

The Wordbank dataset consists of a collection of applications of the MB-CDI. These questionnaires contain zero abstract nouns, 5 abstract verbs and 6 abstract adjectives, but 325 concrete nouns, 48 concrete verbs and 9 concrete adjectives. For this comparison, we have included all abstract words of the dataset. For a comparison between the earliest concrete and abstract words, we have selected the earliest concrete nouns, verbs and adjectives proportional to their share of the dataset, yielding 13 nouns, two verbs and one adjective (please note: eeasuring AoA with equal-sized word type samples would skew concrete AoA higher – concrete verbs and adjectives are acquired later than concrete nouns, but many more concrete nouns (325) than verbs (48) or adjectives (9) are acquired.). We have included all abstract words of the dataset. This sample should be representative of the early concrete and abstract lexicon of 9–30 month-olds. In line with previous research using these data, we consider a word as acquired if > 50% of children in the dataset are reported to produce it (Goodman et al. 2008).

As this is hardly a representative sample of concrete and abstract AoA, especially throughout later years of life, we made the second estimate with all relevant concrete and abstract words in the Kuperman at al. (2012) data. We excluded all personal pronouns, articles, conjunctions, prepositions, quantifiers, interrogative words, negations and auxiliary words. After this exclusion, 5,927 concrete and 5,812 abstract words remained.

### Computational model

We used a BCNN model (Pulvermüller et al. 2021) of cortical areas involved in word, action and object acquisition. The distinguish between model and real cortical areas, we denote model areas with an asterisk. Specifically, we have implemented an auditory processing stream (areas *A1, *AB, *PB), an articulatory motor production stream (areas *M1_i_, *PM_i_, *PF_i_), the dorso-lateral hand-motor production stream (areas *M1_L_, *PM_L_, *PF_L_) and the ventral visual processing stream (areas *V1, *TO, *AT). All areas of the model are connected according to neuroanatomically determined nerve tracts (Tomasello et al. 2018). Each area of the model consists of 625 leaky integrate-and-fire excitatory (e-) and 625 graded response inhibitory (i-) neurons. The inhibitory neurons, together with an area-wide ‘global’ inhibition regulate neural activity. Synaptic weights between neurons change through long-term potentiation as well as hetero- and homosynaptic long-term depression. The complete mathematical description of the model can be found in the Supplementary Materials S1. This model has previously been used to investigate the neural mechanisms behind other semantic phenomena, such as object-action grounding (Carriere et al. 2024; Tomasello et al. 2017, 2018), oscillatory brain responses to words and pseudowords (Garagnani et al. 2016), ‘fast mapping’ of novel wordforms to referents (Constant et al. 2023), concrete and abstract concept encoding (Dobler et al. 2024; Henningsen-Schomers et al. 2023; Henningsen-Schomers and Pulvermüller 2022), and highlighting or suppression of unique features in perceptual processing through the use of proper names or category terms (Nguyen et al. 2024).

### Neural activation patterns

The networks were used to simulate visual perception, action execution, and perception and production of spoken wordforms. To this end, predetermined sets of artificial neurons in primary sensory and motor areas of the network were activated. Activation consisted of 2 time steps of activity for 12 neurons per pattern. Each neuron included in a pattern was thought to encode or represent a specific feature of the entity simulated by the pattern. For example, a given neuron in *A1 represented the acoustic-phonological feature [+ bilabial] of one of the phonemes of a spoken wordform and a *V1 neuron coding for the visual feature [+ round]. What type of information the activity pattern encoded depends on which simulated cortical area it was presented to: activity in *V1 encoded a visual stimulus, but activity in *M1_l_ encoded a hand motor action. Likewise, activity in *A1 corresponded an auditory stimulus, and *M1_i_ an articulatory action. Words were thus simulated through patterns of correlated neural activity in *M1_i_ and *A1, corresponding to hearing one’s own speech near-simultaneously to producing it.

Each instance of a concept – for example one specific hammer – was coded as activation of a set of 12 neurons per primary extrasylvian area. Half of the neurons represented features shared between all instances of hammers, which we call ‘shared neurons’. The other half of the neurons of the pattern was not shared with any other activation pattern and thought to represent unique visual and motor features of a given instance (‘unique neurons’). To account for variability within concepts (Rosch and Mervis 1975), we implemented three instances per concept.

The crucial difference between concrete and abstract concepts in this implementation was the structure of shared features or neurons. While concrete concepts were characterized by features that were shared among all instances (Fig. 1C, D), instances of abstract concepts show pair-wise sharedness: while a beautiful sunrise and the eye of a loved one are both round, *being round* is not a necessary feature for all instances of the concept *BEAUTY*.

The full stimulus set consists of 10 concrete concepts with 3 exemplars each (30 patterns total), 10 abstract concepts with 3 exemplars each (30 patterns total) and 10 wordform patterns.

### Learning concepts and words

Infants and young children may learn a new word for new objects and concepts not already built in their minds, or they may learn words for objects and concepts they already experienced in the past and may have built a neuronal and cognitive representation for. In addition, the wordform may already have been experienced so that a phonological representation may have pre-formed. To capture these different possibilities, we implemented two different learning regimes: Single Stage Learning (Experiment 1, Fig. 2A), during which concept-related and wordform-related patterns were co-presented simultaneously, and Two-Stage Learning (Experiment 2, Fig. 2B), which was split into an initial ‘pre-grounding’ stage, during which concept and wordform patterns were presented separately, and a ‘word-concept mapping’ stage, during which both were co-presented (see Constant et al. 2023).

Across both experiments, training parameters were identical. Stimulation to primary model areas lasted 16 time steps. Between stimulus presentations, primary model areas received uncorrelated white noise activity to simulate background activity in cortex. The duration of the interstimulus interval was determined by the levels of global inhibition in *A1 and *PB, which had to be below a threshold of 0.75 and 0.65, respectively, for the next trial to be initiated (as in Dobler et al. 2024; Henningsen-Schomers et al. 2023; Henningsen-Schomers and Pulvermüller 2022). This prevented persistent neuronal activity of a given trial from influencing subsequent ones.

#### Experiment 1: Single-Stage Learning

In Experiment 1, 24 networks with randomly initialized starting weights were trained per Semantic Type (concrete/abstract) and Learning Condition (Conceptual Learning (without wordform)/Semantic Learning (with wordform)); this resulted in 96 networks overall. Training consisted of 2000 simultaneous trials. Each trial consisted of simultaneous presentation of all extrasylvian activity patterns (*V1 and *M1_L_) in randomized order.

In the Concept Learning condition (without wordform learning), the perisylvian primary areas *A1 and *M1_i_ received uncorrelated ‘noise’ stimulation. In the Semantic Learning condition (with wordform learning), the perisylvian primary areas were instead co-activated with the wordform corresponding to the concept (i.e. a unique hammer is shown in *V1, and the word “hammer” is articulated and heard in *M1_i_ and *A1; see Fig. 2A). Since we implemented 3 instances per concept, and each instance pattern is presented for 2,000 trials, each wordform is presented for 6,000 trials total. This matched the number of trials on the concept-level (3 instances x 2,000 trials).

In the Semantic Learning condition, CAs that were learned from the wordform stimulation and CAs that were learned from sensorimotor concept instances will likely co-activate. However, our analysis targets semantic representations, rather than word representations. Thus, we aimed to exclude neurons specific to wordforms from the analysis. To identify these neurons, we trained 24 wordform control models. They were initialized with the same connectivity as their corresponding Semantic model and were trained with correlated wordform pattern presentation to *A1 and *M1_i_, and uncorrelated white noise input to the extrasylvian primary areas. A CA learned by this model is not grounded in non-linguistic sensorimotor experience and thus exclusively represents semantics-invariant information about the articulatory and acoustic phonological features of a wordform. In our analysis, we excluded all neurons found in these control CAs, which reduced the absolute neuron count. However, this exclusion had no impact on functional measures, as these neurons were functional in the network and contributed to network activity during learning and testing.

#### Experiment 2: Two-Stage Learning

In Experiment 2, 24 models per semantic type (concrete/abstract, 48 models total) were trained in two stages. During the first stage, in each trial the model was either stimulated with a correlated pair of extrasylvian activation patterns (*V1 and *M1_L_) *or* a correlated pair of perisylvian activation patterns (*A1 and *M1_i_), respectively modelling concept and wordform learning (as illustrated in Fig. 2B). This process was repeated for 2,000 training trials per stimulus. Compared to Experiment 1, the wordform presentation frequency now matched that of each instance, not the overall number of presentations of all three conceptual instances together. Pilot simulations indicated that this adjustment was necessary: since the neurons in wordform activation patterns were more highly correlated than the unique or two-way shared feature neurons in conceptual instance patterns, the resulting wordform CAs outcompeted concept CAs when the number of presentations matched Single Stage Learning. Rather than changing the stimuli between the two experiments, we have decided to decrease the number of presentations for wordform activation patterns.

No wordform control models were trained for Two-Stage Learning, as the training paradigm ensures that wordform CAs remained disconnected from concept CAs. Instead, the CAs derived from wordform testing at the end of the first stage were used as the wordform control.

### Assessment of learning results

During and after training, models were tested by using the same activation patterns used during training to measure the network-wide activity in response to these patterns as a result of learning. Stimulation lasted for two time steps and network responses were recorded during 28 subsequent time steps. For Conceptual and Semantic Learning models, we stimulated *V1 and *M1_L_. For Wordform Control models, we stimulated *A1 and *M1_i_. The synaptic weights of the models were fixed during testing, so that no further learning could occur. Throughout testing, no white noise background activity was provided for Single Stage Learning, as doing so would lead to exaggerated model activity during early training trials, when no reliable stable synaptic weights were reached yet. For Two-Stage Learning, white noise background activity (noise parameter = 8) was provided. Prior to stimulation, we recorded a baseline of 10 time steps of network activity. Between testing trials, model activity was reset by setting the membrane potential of all neurons to 0.

For Single Stage Learning, the testing interval was every 20 training trials to track the long-term acquisition trajectory. For Two-Stage Learning, the testing interval in the first stage was every 50 training trials. To assess word-concept mapping, we tested after each of the first 50 learning trials in both Single Stage Learning, and the second stage of Two-Stage Learning, as previous research using this model has shown a fast mapping of wordform to semantics within the first 20 trials (Constant et al. 2023) and we thus expected main effects to be found during early trials. Pilot tests showed that CAs stabilize after approximately 150 trials in this model, therefore we tested every 10 trials until trial 100, and then every 50 trials until trial 500.

To assess the learned CAs, we recorded the activity of all neurons of the network following stimulation of each activity pattern. We classified responsive neurons as part of the CA if their dynamic estimated firing rate reached 75% of the maximal firing rate in their area for at least two time steps. For CAs from extrasylvian pattern stimulation we additionally classified each active neuron according to whether it was activated by only one single instance-related pattern (“unique” or “instance-specific neurons”) or by two or all of a concept’s instances (“shared” or “conceptual/semantic neurons”).

To assess word-concept mapping, we presented the extrasylvian conceptual instance activity patterns in *V1 and *M1_L_ and identified the responsive CA. Then, we stimulated wordform activation patterns in *A1, corresponding to hearing the word. conducted a simulation of auditory wordform comprehension, wherein we presented the wordform activity pattern to *A1. We measured how many neurons in the concept CA responded after auditory stimulation and considered a concept CA as linked to the wordform if more than 30% of the concept CA responded.

We measured the following structural and functional CA correlates: (1) wordform-to-concept mapping (2) reverberation period (number of consecutive time steps above the baseline firing rate), (3) number of shared and unique CA neurons. measure (1) tested whether the models successfully linked wordform and concept. Measure (2) assessed the functional viability of the CA: if a given neuronal assembly would not remain active for longer than the standard response of the network to stimulation, it is an unlikely correlate for cognitive processes. Finally, measure (3) assessed the internal structure of the CA, i.e. whether it was predominantly made up of neurons that respond to only one (unique), multiple (two-way shared) or all instances (three-way shared) of the same concept.

All results are based on a two-way repeated measures ANOVA with the factors Semantic Type (concrete/abstract) and Training Trial. The analyses for (1) and (2) only include data from the extrasylvian areas of the model. To localize effects, we additionally conducted a three-way repeated measures ANOVA with the within-factors Semantic Type (concrete/abstract), Area (Primary/Secondary/Central) and Training Trial, which are reported in the Supplementary Materials S3.

If the ANOVA was significant, we conducted post-hoc pairwise two-tailed t-tests between concrete and abstract concepts for each Training Trial. All t-test results have been Bonferroni-corrected for the number of Training Trials (Single stage learning: 100, Two-stage learning, pre-grounding stage: 25; Experiment 2, word-concept mapping stage: 73). All non-significant Training Trials are highlighted with a grey background in the following Figures.

For Single Stage Learning, 5 networks were removed prior to statistical analysis due to merging of representations in either the concrete, abstract or the wordform control condition. Merging meant that multiple CAs would coactivate (i.e. multiple wordform CAs or instances from different concepts) with excessive neuronal activity, making them impossible to analyse. An additional 3 networks were removed for producing outlier CA sizes (+/−1.5*IQR). For Two-Stage Learning, no networks were removed.

## Results

### AoA estimation

Developmental trajectories show that concrete nouns, verbs and adjectives tend to be acquired earlier than abstract ones (Fig. 3A). Concrete words had a mean AoA of 20.2 +/- 1.3 months, abstract words of 26.4 +/- 2.3 months; the difference was significant with a Two Sample t-test (t(23)=−8.5, *p* < 0.05, CI95=[−7.67, −4.67]) (see Fig. 3B). The earliest acquired abstract words showed a 30.5% (6.2 months) delay relative to concrete ones.

In the Kuperman et al. (2012) ratings, concrete words were rated to have a mean AoA of 8.6 +/- 2.7 years, but abstract words 11.3 +/- 2.3 years; the difference was significant with a Welch Two Sample t-test (t(11,480)=−59.06, *p* < 0.05, CI95=[−2.83, −2.65]) (see Fig. 3C). While the larger sample implies a greater absolute difference, the relative delay was 32.0%. Thus, both simultaneous early acquisition and lifetime retrospective AoA estimates indicate a relative delay for abstract words of approximately 30%.

### Experiment 1: Single Stage Learning

#### Word-concept mapping

During Semantic Learning, we tested word-concept mapping in early trials. There was a significant difference for ‘AoA’ (the trial at which more than 50% of models linked the wordform and concept CA) between concrete and abstract concepts (Two-Sample t-test, t(18)=−3.79, *p* < 0.01, CI95=[−16.16, −4.64]) (see Fig. 4). Concrete concept CAs were linked to their respective wordform earlier than abstract ones (concrete: mean = 41.0, SD = 4.1; abstract: mean = 51.4, SD = 7.6). The relative difference between AoAs was 22.5%, lower than the estimate derived from empirical data. We will now evaluate whether there were differences in concept CA function and structure in both conditions and consider how these differences lead to the delay.

#### Reverberation

**Fig. 5:**
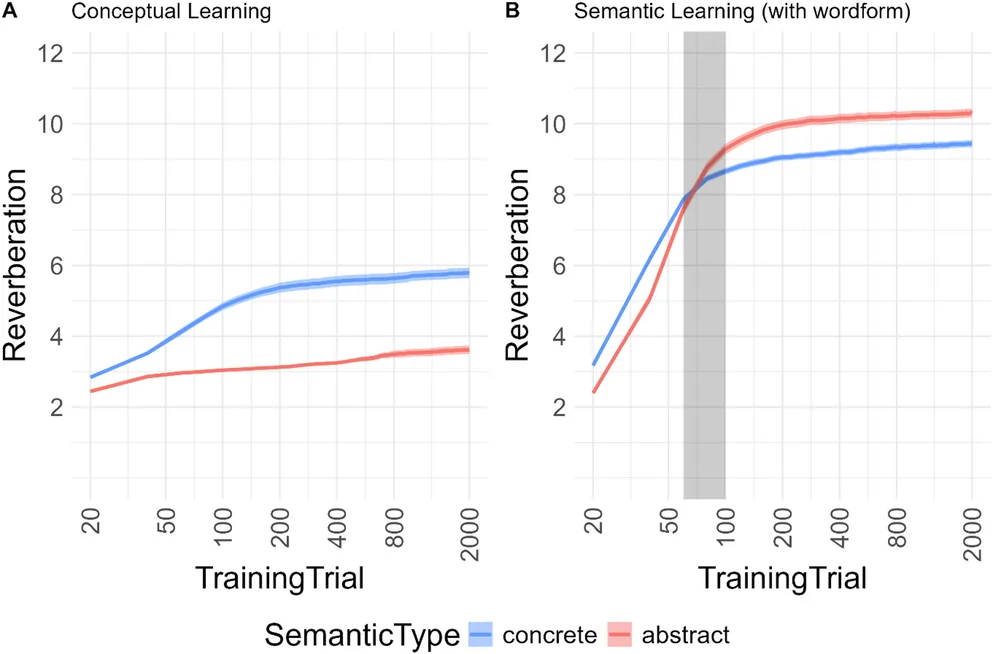
**A** CA reverberation throughout the Conceptual Learning condition. **B** CA reverberation throughout the Semantic Learning condition. While reverberation stops increasing after 200 training trials in both conditions, the rise in reverberation is steeper for Semantic Learning

During Conceptual Learning, we found a significant interaction between Semantic Type x Training Trial for reverberation (F(99, 1,485) = 23.75, *p* < 0.001). Post-hoc t-tests showed a significant difference between concrete and abstract concepts for all Training Trials (all *p* < 0.001). The interaction was also significant during Semantic Learning (F(99, 1,485) = 63.51, *p* < 0.001), however, post-hoc t-tests showed no significant difference between trials 60–100. (see Fig. 5). By-area analysis showed that the first trials without significant difference occur in Central model areas (*AT, *PF_L_), which are densely connected to perisylvian areas (see Supplementary Materials S3). We detected an upwards trend for reverberation in the abstract Conceptual Learning condition. To test whether they could reach the reverberation period of concrete models, we extended training to 10,000 training trials. There was still a significant difference in reverberation time between concrete (mean: 5.87, SD = 0.62) and abstract concepts (mean: 3.91, SD = 0.58) at trial 10,000 (t(14) = 8.99, *p* < 0.001, CI95=[1.49, 2.44]; see Supplementary Materials S2).

Reverberation increased steadily for concrete circuits during Conceptual Learning, but not for abstract ones. Reverberation is both a measure of CA functionality and a beneficial property for Hebbian learning: the more often neurons of a CA fire together during a trial, the more frequently LTP strengthens their synaptic connections. Low reverberation for abstract concepts thus suggests major difficulties in acquiring a stable neural representation of the inputs. During Semantic Learning, reverberation increased for abstract concept CAs already during early trials, followed by an even steeper increase starting at trial 40. These results imply an early facilitation of abstract concept learning by co-presentation of a wordform. Next, we will investigate how this drastic change in functionality arises from structural changes within the neuronal circuits.

#### Structural changes

**Fig. 6:**
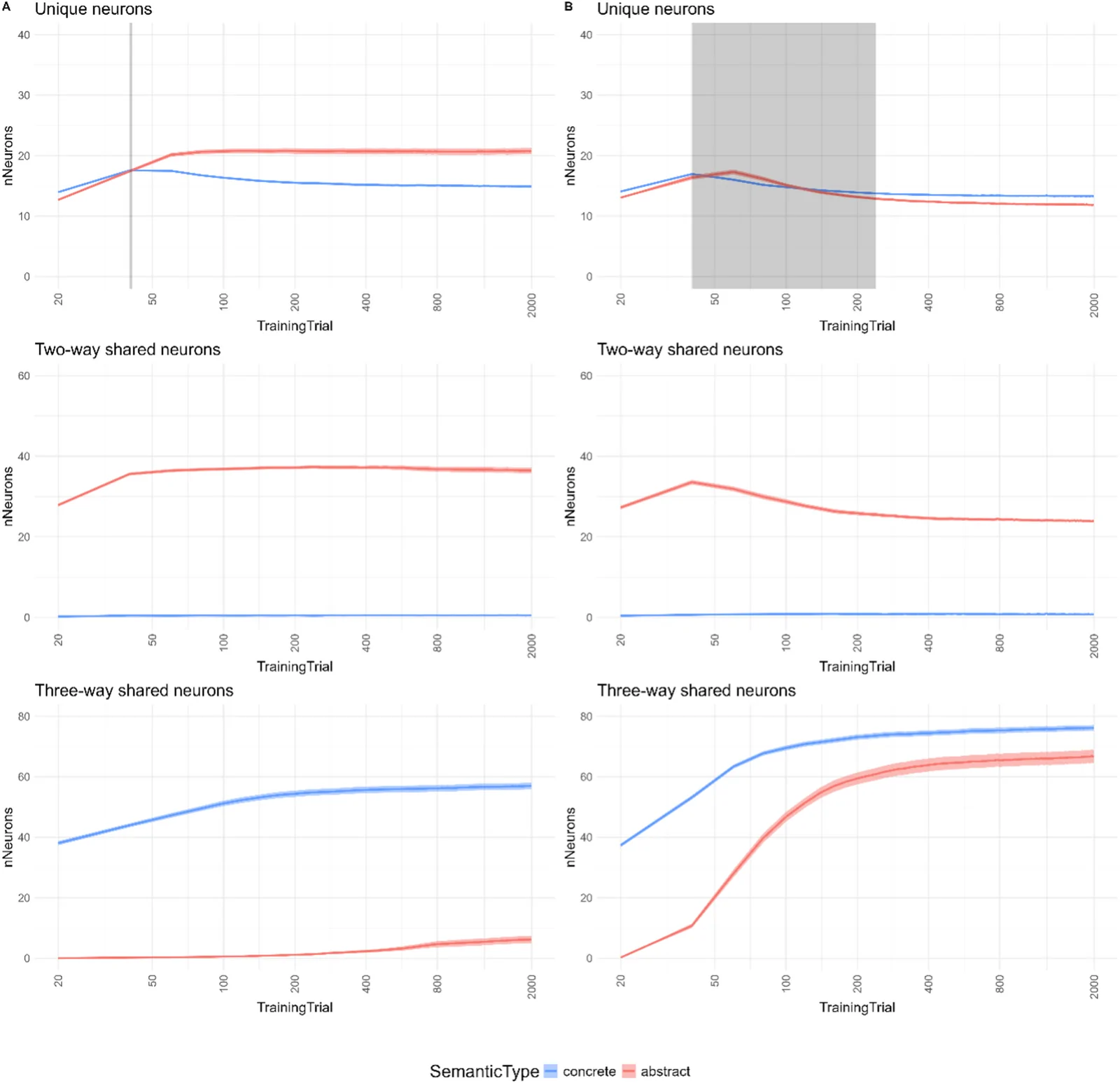
Changes in CA composition throughout training. **A** Number of unique (top), two-way shared (middle) and three-way shared (bottom) neurons during Conceptual Learning. **B** Number of unique (top), two-way shared (middle) and three-way shared (bottom) neurons during Semantic Learning (with wordform). Concrete CAs are displayed in blue, abstract in red. The grey background highlights trials with non-significant differences between concepts

During Conceptual Learning, we found a significant interaction between Semantic Type x Training Trial for the number of unique (F(99, 1,485) = 122.9, *p* < 0.001), two-way shared (F(99, 1,485) = 29.9, *p* < 0.001) and three-way shared neurons (F(99, 1,485) = 14.6, *p* < 0.001). Post-hoc t-tests showed a significant difference between concrete and abstract concepts for all Training Trials for all neuron types, except for unique neurons at trial 40 (all other *p* < 0.05) (see Fig. 6A).

During Semantic Learning, we found a significant interaction between Semantic Type x Training Trial for the number of unique (F(99, 1,485) = 20.8, *p* < 0.001), two-way shared (F(99, 1,485) = 90, *p* < 0.001) and three-way shared (F(99, 1,485) = 109.8, *p* < 0.001) neurons. Post-hoc t-tests showed a significant difference between concrete and abstract concepts for all Training Trials for all neuron types, except for unique neurons at trials 40–240 (all other *p* < 0.05) (see Fig. 6B).

The neuronal response to instances of concrete concepts during Conceptual Learning gradually recruited less unique neurons, and more three-way shared neurons. Meanwhile, instances of abstract concepts elicited a response of predominantly unique and two-way shared neurons, which stabilized early in training. Additional training trials did not lead to a significant development of concept-wide shared neurons. During Semantic Learning, concrete concept CAs followed a similar, but exaggerated trajectory as in Conceptual Learning. The response to abstract concept instances, however, changed. Rather than the circuits stabilizing early, the number of unique and two-way shared neurons decreased, while the number of three-way shared neurons increased. In short, the developmental trajectory of abstract concept representations became similar to that of concrete ones. Notably, the increase of three-way shared neurons coincided with the initial, and then steeper increase in reverberation discussed above (see Fig. 5B), implying that three-way shared neurons supported longer reverberation times.

### Experiment 2: Two-Stage Learning

#### Word-concept mapping

**Fig. 7:**
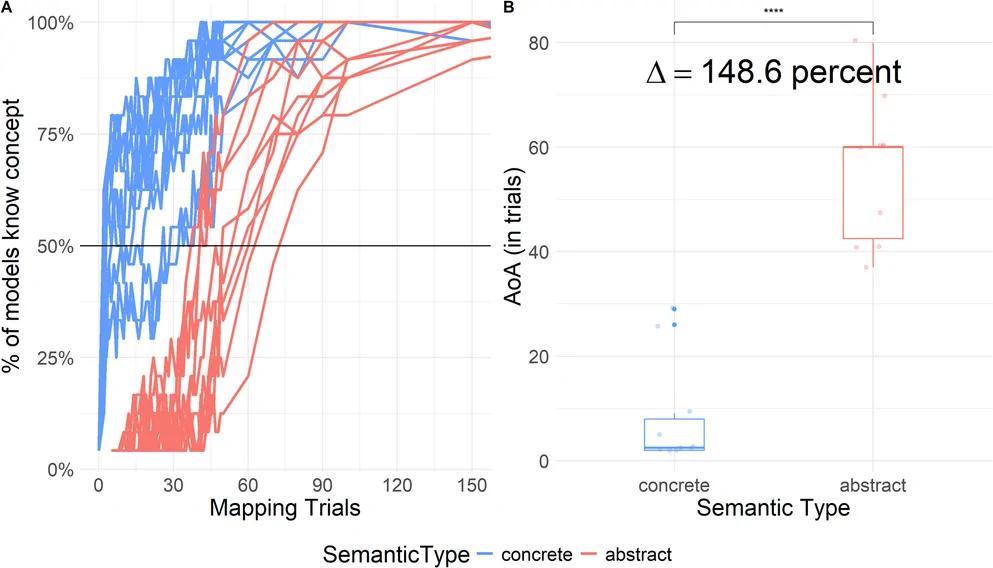
**A** Developmental trajectories of word-concept mapping. In line with trajectories reported in AoA literature, we plot a population-wide acquisition average against ‘model age’ (number of mapping trials). Each line corresponds to a single concrete (blue) or abstract (red) concept. We consider a word mapped to its concept within a model if auditory stimulation elicits activity in > = 30% of the extrasylvian concept cell assembly. For the AoA estimation, we consider it acquired at the trial when > = 50% of all models have successfully mapped concept and word. **B** AoA (in trials) for concrete and abstract concepts

Like in Single Stage learning, we tested word-concept mapping in early trials of the mapping stage. There was a significant difference for AoA between concrete and abstract concepts (Two Sample t-test, t(18)=−8.64, *p* < 0.001, CI95=[−58.92, −35.88]) (see Fig. 7B). Concrete concept CAs were linked to their respective wordform earlier than abstract ones (concrete: M = 8.2, SD = 10.4; abstract: M = 55.6, SD = 13.8). The relative difference between AoAs was 148.6%, higher than the estimate derived from empirical data. Post-hoc t-tests were significant until Trial 150 (all *p* < 0.05) (see Fig. 7A).

Comparing the two-stage learning AoAs to single-stage learning, we found that the pre-grounding of concepts facilitated word-concept mapping for concrete concepts (Two Sample t-test, t(18) = 9.3, *p* < 0.001, CI95=[25.36, 40.24]) but had no impact on abstract concept mapping (t(18)=−0.83, *p* = 0.41, CI95=[−14.71, 6.31]). We will now investigate which functional and structural properties of concrete and abstract neuronal circuits before and after mapping can explain that effect.

#### Reverberation

**Fig. 8:**
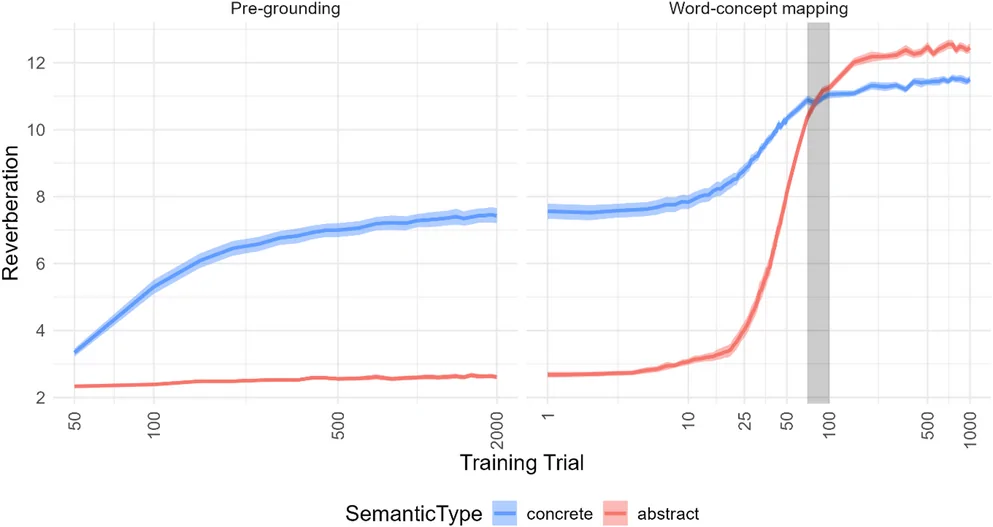
Reverberation throughout both training stages. Concrete concepts CAs (in blue) reach ~ 7.5 timesteps of reverberation during pre-grounding, while abstract ones (in red) reach ~ 2.75. During word-concept mapping, reverberation improves for both types of CA between trials 10–100. The absolute improvement is larger for abstract CAs

The interaction between Semantic Type x Training Trial for reverberation was significant during the pre-grounding phase (F(24, 552) = 138.8, *p* < 0.001) and the word-concept mapping phase (F(72, 1656) = 213.3, *p* < 0.001). During pre-grounding, all post-hoc t-tests were significant (*p* < 0.01). During word-concept mapping, results were significant for all trials (*p* < 0.05), except 70–100 (see Fig. 8).

In the first phase, models learned concept and wordform activation patterns separately. The resulting reverberation curves were comparable to the Conceptual Learning condition: concrete concept activation patterns lead to reverberant cell assemblies, abstract ones to neuronal circuits that could not sustain activity. During word-concept mapping, the abstract neuronal circuits increased in reverberation only after 10 training trials, and then sharply increased from 3 to 10 time steps of reverberation within the next 60 trials. How did the underlying CA structure change?

#### Structural changes

**Fig. 9:**
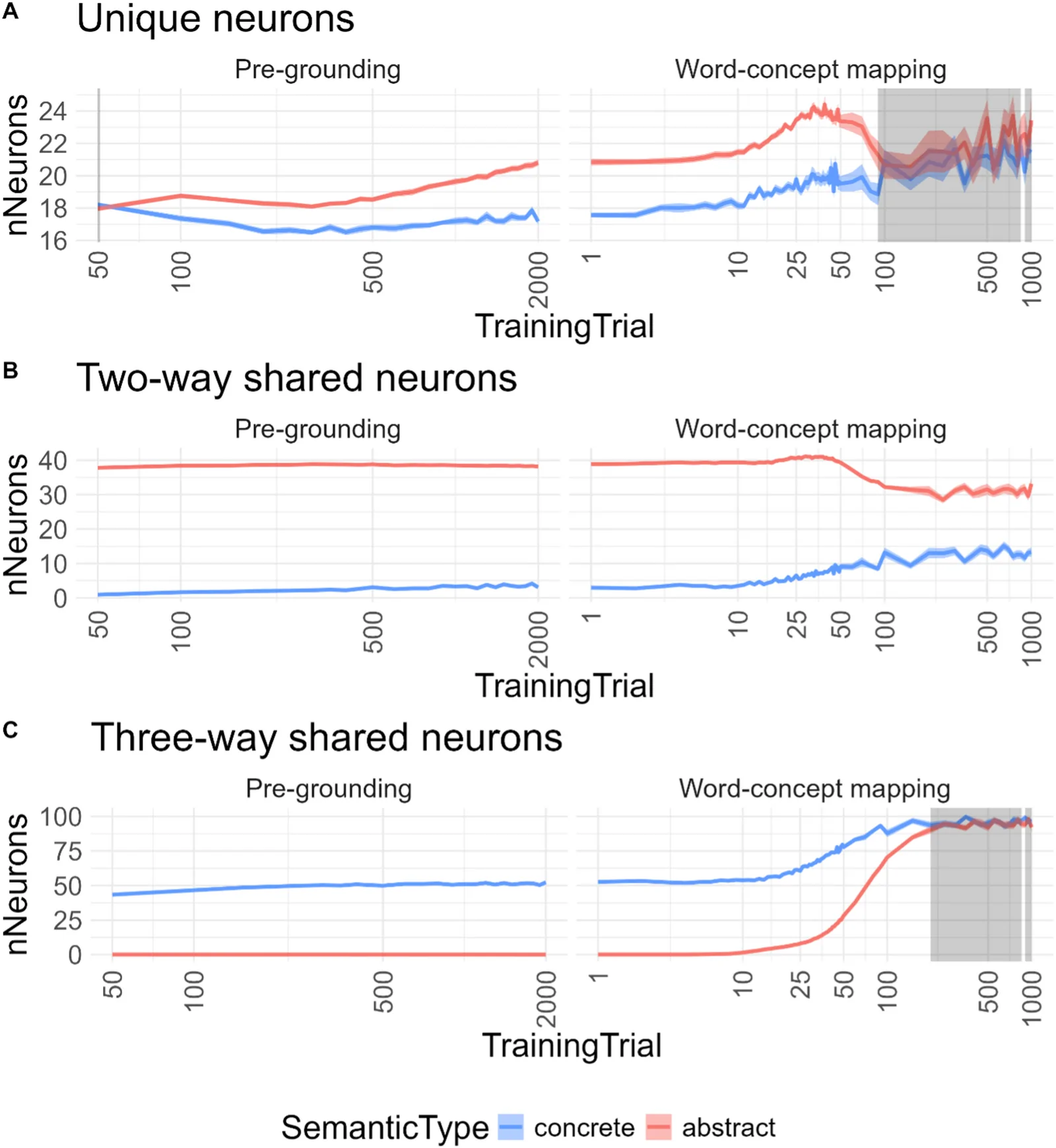
Changes in CA composition throughout training. Number of **A** unique, **B** two-way shared and **C** three-way shared neurons during Conceptual Learning. Concrete CAs are displayed in blue, abstract in red. The grey background highlights trials with non-significant differences between concepts

During pre-grounding, we found a significant interaction between Semantic Type x Training Trial for the number of unique (F(24, 552) = 19.88, *p* < 0.001), two-way shared (F(24, 552) = 6.91, *p* < 0.001) and three-way shared neurons (F(24, 552) = 15.96, *p* < 0.001). Post-hoc t-tests showed a significant difference between concrete and abstract concepts for all Training Trials for unique, two-way shared and three-way shared neurons (except: unique neurons at trial 50, t(23) = 1.47, *p* = 0.16; all other *p* < 0.001) (see Fig. 9).

During word-concept mapping, we found a significant interaction between Semantic Type x Training Trial for the number of unique (F(72, 1,656) = 3.04, *p* < 0.001), two-way shared (F(72, 1,656) = 43.79, *p* < 0.01) and three-way shared neurons (F(72, 1,656) = 131.62, *p* < 0.001). Post-hoc t-tests showed non-significant Trials from trial 90 onwards for unique neurons. The difference between the number of two-way shared neurons remained significant throughout. Finally, the number of three-way shared neurons ceased being significantly different starting at trial 200. By-area analysis showed that this was driven by changes in the central extrasylvian areas *AT and *PF_L_ (see Supplementary Materials S3).

The results of the pre-grounding stage aligned with Conceptual Learning: neuronal circuits learned from concrete activation patterns formed predominantly three-way shared neurons, with the number of unique neurons decreasing throughout training. Those learned from abstract activation patterns however consisted predominantly of unique and two-way shared neurons. Three-way shared neurons only developed during word-concept mapping, starting at 10 training trials and rapidly increasing until trial 150. This development paralleled the increase in reverberation time, and the time course of words being successfully mapped to abstract concepts. As in Semantic Learning, the number of unique and two-way shared neurons decreased as the number of three-way shared neurons increase, indicating structural changes to the CA.

The number of unique neurons increased for concrete concepts in the second stage, implying an improved item-level representation compared to Single Stage learning.

## Discussion

We simulated abstract and concrete word learning in a BCNN model to assess how differences in concept structure affect AoA. We implemented two avenues of acquisition, to capture concept learning with and without verbal support, and word-concept mapping with and without previous concept learning. In Single Stage Learning, concepts and their corresponding wordforms were learned simultaneously, whereas Two-Stage Learning started with a pre-grounding phase, in which concepts and wordforms were acquired separately. Concept instances and words were only co-presented in the second stage.

Abstract words linked with their corresponding concept later than concrete ones in both experiments, replicating the acquisition delay seen in human data. Importantly, this delay cannot be explained by typical in vivo confounds: the word activity patterns used for concrete and abstract words were identical, ruling out psycholinguistic effects. Both concrete and abstract words were presented with the same frequency and had identical chances of co-occurring with a relevant concept instance. Since we simulated direct, rather than indirect grounding, an existent more concrete vocabulary was not required for abstract word learning. Thus, the delay arose only from differences in concept structure.

By analysing the structure and function of the neural representations in the model, we can theorize what might cause this delay on a neuromechanistic level outside of the aforementioned factors.

### Conceptual representations formed pre-verbally for concrete, not abstract concepts

Conceptual representations of concrete concepts formed at the neural level as CAs. These CAs comprised shared neurons belonging to all representations of conceptual instances (such as different variants of HAMMERs) and formed as a consequence of processing different instances (Figs. 7A and 9 ‘pre-grounding’). Neurons responding to shared features of these instances frequently co-activated, thus forming strong synaptic connections through LTP. This kernel of highly connected shared neurons contributed to a comparatively long reverberation time of ~ 6–7.5 simulation time steps for concrete concept CAs (Figs. 6A and 8 ‘pre-grounding’).

Instead of reverberant representations, the processing of instances of abstract concepts only lead to the formation of short-lived (4 time steps in Single Stage Learning, Figs. 2 and 5A time steps in pre-grounding Two-Stage Learning, Fig. 8) neural responses. The responses comprised of neurons only shared by several, not all instance representations (Figs. 7A and 9 ‘pre-grounding’). These results are in line with the acquisition of certain concepts by pre-verbal infants (Mandler 2004; Perszyk and Waxman 2018) or non-verbal species (Pearce 2008; Pusch et al. 2023), and with previous neurocomputational simulations (Dobler et al. 2024; Henningsen-Schomers and Pulvermüller 2022).

After introducing a wordform – either from the start, in the Semantic Learning condition, or during the word-concept mapping trials – we observed characteristic changes in the structural makeup of the neuronal correlates of the learned concepts. Specifically, the number of three-way shared neurons – neurons which respond to any concept instance – increased. Since we removed all wordform-specific neurons from the analysis, these novel three-way shared neurons could not to be attributed to the wordform CA alone and only emerged through semantic learning of both a concept and a wordform.

For concrete concepts, this meant a quantitative change in structure: concrete CAs already contained three-way shared neurons in the Conceptual Learning condition, and during the pre-grounding stage. For abstract concepts, however, the emergence of concept-level neurons was a qualitative change. Without a wordform, the models did not consolidate the neuronal response to the individual instances into a shared, concept-wide CA. During Semantic Learning and the word-concept mapping stage of Two-Stage Learning, the developmental trajectories of abstract CAs became similar to those of concrete ones: during early trials, the number of instance-specific neurons increased, but throughout training, these neurons became responsive to all instances.

These structural changes preceded changes in functionality. Reverberation time increased only after a substantial number of three-way shared neurons had formed – and word-concept mapping only begun once the CA remained active for long enough.

### A neuromechanistic explanation of abstract word learning

A comparison between the model AoAs in Single Stage and Two-Stage learning showed that concrete words were acquired earlier in Two-Stage learning, linking to the correct concept CA in less than 10 trials. Meanwhile, the AoA of abstract words did not significantly change between experiments. This suggests that linking abstract concepts to wordforms was equally difficult in both experiments, while pre-grounding the concept and word separately facilitated concrete word-concept mapping.

Rapid acquisition of concrete words is in line with behavioural (Carey and Bartlett 1978), neurophysiological (Friedrich and Friederici 2011; Hofstetter et al. 2017) and computational studies of fast-mapping (Constant et al. 2023). The structural and functional differences we found during concrete and abstract concept acquisition provide a potential neuromechanistic explanation as to why abstract words are harder to learn, even if all other psycholinguistic and cognitive factors were the same for concrete and abstract words.

The full overlap structure of concept features for concrete concepts lead to the formation of strongly connected, reverberant CAs in extrasylvian model areas through LTP. Thus, a functional neuronal correlate already existed pre-verbally, meaning that concrete word learning could be described as ‘labelling’ an already existing concept. Linking concept and wordform CAs was supported by the correlation of its auditory and articulatory features with the sensorimotor concept features. For example, if all hammers shared the visual features of being a long stick with a stout head, and all hammers were always called ‘hammer’ – as in this simulation – the correlation would be *p* = 1 between the visual feature neurons and the auditory and articulatory feature neurons for the word (see Fig. 1C). This high correlation, together with longer reverberation time, allowed for the rapid formation of strong synaptic connections between the extrasylvian pre-verbal concept CA and the perisylvian wordform CA.

In contrast, the family resemblance structure of abstract concept features did not allow for the formation of reverberant neuronal correlates. With no pre-verbal conceptual representation, the mechanism of abstract word learning cannot be described as ‘labelling’ within the model. Instead, the concept itself was acquired through co-presentation with the wordform during Semantic Learning. Again, the neuromechanistic explanation of this effect relies on feature correlation between sensorimotor and linguistic features (Pulvermüller 2013): while the correlation between any two sets of shared sensorimotor feature neurons was *p* = 1/3, the correlation between a set of sensorimotor feature neurons and the wordform feature neurons was *p* = 2/3. For example, the feature [round] might be present in two instances of beautiful things: the eye of a loved one, and the sunrise (see Fig. 1C). During Conceptual Learning, this feature co-occurred with the feature [in motion] one out of three times, and the feature [human] one out of three times. With a wordform, however, [round] occurred with the word ‘beautiful’ both for the sunset and the eye – two out of three times. As such, the previously two-way shared neurons could bind onto the wordform CA through LTP. This lead to them co-activating whenever the wordform CA becomes active – for example, when the next concept instance, a dance, is called ‘beautiful’ – and thus strengthen the synaptic connection between previously disconnected neuronal populations. After sufficient training trials, the extrasylvian concept CA has been restructured to contain a large number of concept-level neurons – and the word-concept mapping was successful.

Crucially, the model predicted that this linking – both the ‘labelling’ of concrete concepts, and the wordform-induced restructuring of abstract concepts – is driven by cortical areas that are central to the neural architecture and connect perisylvian and extrasylvian sensory processing and motor action streams (see also Garagnani and Pulvermüller 2016; Nguyen et al. 2024; Tomasello et al. 2017).

A caveat to the validity of the results of the Two-Stage Learning experiment is the discrepancy between the simulated relative AoA difference and the one estimated from human data (see Fig. 4). We calculated this difference for mapping trials, without taking the pre-grounding phase into account. However, that is a poor comparison to human AoA data: AoAs begin at birth and thus encompass the non-semantic ‘pre-grounding’ phase. If we switched to word-concept mapping as soon as pre-grounding CAs were stable (around trial 150), we would estimate the total AoAs to be 155 trials for concrete, and 210 trials for abstract concepts (a 30.14% relative difference, in line with our human estimations).

We decided to simulate a single contribution of language to concept learning, namely additional invariant features that are independent from other modalities. However, concept and word learning are far more complex in vivo. Language is far more than individual words, with conversations providing additional context for concepts throughout acquisition (Borghi et al. 2025; Chang and Deák 2020; Della Rosa et al. 2010; Hoffman et al. 2018; Pexman et al. 2023). Additionally, ‘abstract concepts’ span a wide range of potential semantic domains, from emotions over social interactions to metaphysical concepts (Ciaccio et al. 2025; Villani et al. 2019), not all of which can be adequately operationalized through feature sharing. Thus, the results reported here should be interpreted with caution: they do not imply that associative word-concept learning is the exclusive, or even the main mechanism of abstract concept acquisition. Instead, the results show that even if abstract words had the most level playing field with concrete words – namely psycholinguistic feature matching, equally frequent co-occurrence with a potential referent, the same exclusive learning mechanism for both types of word, no differences in grounding modalities – they would still be acquired with a delay compared to concrete words.

### Language as a direct grounding modality of abstract words

Prevalent theories about abstract semantics in the brain focus on the role language plays. Dual Coding Theory (Paivio 1990) posits that abstract semantics are based on logogen-to-logogen associations, a theoretical account that could explain abstract word learning through distributional semantics or indirect grounding. While these effects are well-documented (Stramandinoli et al. 2017; Wiemer-Hastings and Xu 2005; Zdrazilova and Pexman 2013; Zwaan 2016), they fail to address a crucial problem: often, the verbal explanations given for an abstract concept rely on further abstract concepts. To illustrate, the Oxford Learner’s Dictionary defines a ‘concept’ as “an *idea* or *principle* that is connected with something *abstract*”. Rather than assuming a long chain of indirect grounding until a grounded, meaningful term can be found, it might be prudent to instead consider an avenue of direct grounding of abstract words as the simplest mechanistic explanation.

The reported simulations have shown that the issue with generalizing dissimilar instances into a concept might partially lie in a lack of shared, invariant features. Using the same word in the context of these dissimilar instances can solve this problem by providing invariant features in the auditory and articulatory modality. Indeed, language is a prime candidate to supply such additional information: due to their symbolic nature, words can be used in a variety of contexts by experienced speakers, and speaker community conventions constrain word usage to specific situations, ensuring the invariance required for abstract word acquisition. In this view, spoken language could be its own grounding modality.

### BCNN predictions on concept learning trajectories in different brain regions

Due to the implemented brain constraints in the BCNN, in particular the explicit simulation of specific brain areas, the BCNN makes various explicit and interpretable predictions about the role of the simulated brain areas during word-concept learning. These should be validated in future empirical research to assess whether the model indeed captures relevant neuronal mechanisms and dynamics of the process. First, the model predicted different trajectories of CA formation for concrete and abstract concepts without wordforms. While concrete concepts were represented as large and reverberant, abstract concepts did not elicit a meaningful concept-wide neural response. However, once wordforms were introduced, both CA size and reverberation changed significantly for concrete and abstract concepts. The reverberation time increased for concrete concepts, implying that working memory is enhanced even for highly similar sensorimotor experiences if they were tagged with a wordform. Notably, this enhancement in working memory was not exclusive to perisylvian areas, but also extended to so-called ‘semantic hubs’ like the anterior temporal lobe and the dorsolateral prefrontal cortex, and into primary and secondary visual and motor areas (see Supplementary Materials).

Additionally, the model predicted that the first areas outside of the perisylvian core language system affected by word-concept learning were the ‘semantic hubs’ *AT and *PF_L_. Specifically, the change might be reflected in a longer lasting neuronal response throughout the area, as well as a stronger response to a larger number of different instances of the same concept.

### Limitations

There are four main limitations to this study: first, the concepts fall on extreme ends of the concrete-abstract spectrum and are rather simplistic. Simulations of concept learning should include prototypical and peripheral instances, between-concept relationships and a larger number of instances. Second, the model does not include all cortical and subcortical regions that are implicated in processing abstract meaning, especially emotion words. Both expanding the stimulus set and the model were unfeasible for present work due to technical limitations in simulation speed. However, the model utilized in this study has recently been implemented in the NEST simulation framework (Gewaltig and Diesmann 2007), which improved simulation speed by a factor of 6 (Carriere et al. 2026) – making it feasible to address these limitations in future work. Third, as with any neurocomputational modelling study, we cannot claim with absolute certainty that the model captured all necessary brain properties to qualitatively simulate human processes of word-concept learning. However, due to the multiple brain constraints implemented in the model, in addition to its validation on one-to-one word-entity learning, we are optimistic that the BCN predictions can be validated on empirical brain data in future research.

Finally, as discussed above, the specific focus on word-concept associations does not allow for inferences on other avenues of abstract concept learning. However, grounding of abstract words in direct experiences has long been ruled out as unfeasible, but these simulation results imply that it could indeed be possible.

## Conclusion

Abstract words tend to be acquired later than concrete words. We conducted a brain-constrained neurocomputational simulation study to identify putative brain mechanisms underlying this delay based exclusively on Hebbian and anti-Hebbian learning. The brain-like networks formed stable conceptual representations in the form of cell assembly circuits for concrete concepts pre-verbally, but not for abstract ones. Introducing a word form with concept learning led to a direct link between the word cell assembly and the concrete concept CA. Linking was impossible for abstract concepts, since no neuronal representation formed pre-verbally. However, the wordform provided crucial situation-invariant features, that allowed for a stable cell assembly to form for abstract concepts as well. Thus, the delay in acquisition of more abstract words might be due to the neural encoding of abstract concepts having to develop co-ontologically to the word-concept mapping. Finally, the model predicted an early role of densely connected ‘semantic hub’ areas in the representation of abstract concepts, which might reflect a bigger role of linguistic features on abstract over concrete concepts.

## Supplementary Information

Below is the link to the electronic supplementary material.

Supplementary Material 1

## Data Availability

All stimuli, data and code for statistical analyses used in this study can be found online (https://osf.io/ukcdv).
