# Supplementary material for: What is so hard about abstract words, anyways? A neuromechanistic explanation using brain-constrained neural network models
Source: Cogn Neurodyn. 2026 Sep 24;20(1):186. doi: 10.1007/s11571-026-10542-z (PMC13612806; doi:10.1007/s11571-026-10542-z)
Supplement: Supplementary file 1 [file 11571_2026_10542_MOESM1_ESM.docx]

# Supplementary materials

**Article Title:** What is so hard about abstract words, anyways? A neuromechanistic explanation using brain-constrained neural network models
**Authors:** Fynn R. Dobler, Lorenzo Stroppa, Friedemann Pulvermüller

**Corresponding author:** Fynn R. Dobler ([fynn.dobler@fu-berlin.de](mailto:fynn.dobler@fu-berlin.de)), Brain Language Laboratory, Department of Philosophy and Humanities, WE 4, Freie Universität Berlin

## Appendix S1: Structure and Function of the Spiking Neuron Model^[1]^

Each of the 12 model areas consists of 625 *e*-cells and 625 *i*-cells, where *e*-cells model single pyramidal spiking neurons, and *i*-cells model a cluster of inhibitory interneurons located within the same cortical column as the corresponding *e*-cell (Eggert & van Hemmen, 2000; Wilson & Cowan, 1972). The state of each cell $x$ at time $t$ is defined by its membrane potential $V\left( x,t \right)$, which is specified by the following equation:

|  | $\tau\cdot\frac{dV\left( x,t \right)}{dt}=-V\left( x,t \right)+k_{1}\left( V_{In}\left( x,t \right)+k_{2}\eta\left( x,t \right) \right)$ | ( 1 ) |
| --- | --- | --- |

Where $V_{In}\left( x,t \right)$ is the net input to cell $x$ at time $t$ (sum of all inhibitory and excitatory potentials), $\tau$ is the time constant of the membrane, $k_{1}$ and $k_{2}$ are scaling constants and $\eta\left( x,t \right)$ is a white noise process with a uniform distribution over $\left[ -0.5, 0.5 \right]$. For inhibitory neurons, $k_{2}=0$. Thus, noise was inherently present in all excitatory, but not inhibitory, neurons. $V_{In}\left( x,t \right)$ is defined as follows:

|  | $V_{In}\left( \text{x},\text{t} \right)=-k_{G}\omega_{\text{G}}\left( A_{x},t \right)+\sum_{\forall\text{y}} \text{w}_{\text{x},\text{y}}\cdot\phi\left( y,t \right)$ | ( 2 ) |
| --- | --- | --- |

Where $y$ is a another cell in the network, $\text{w}_{\text{x},\text{y}}$ is the weight of the synapse between cell $y$ and cell $x$, $\phi\left( y,t \right)$ is $y$’s output at time $t$ (see Equation ( 3 )), $\omega_{\text{G}}\left( A_{x},t \right)$ is the global inhibition for the area $A$ in which cell $x$ is located at time $t$ and $k_{G}$ is a scaling constants for global inhibition. Note that global inhibition is only applied to excitatory neurons. Synapses between inhibitory and excitatory cells are implemented as having a negative value.

The output of an *e*-cell at time $t$ is defined as:

|  | $\phi\left( e,t \right)=\left\{ \begin{aligned} 1,\text{if }V\left( e,t \right)-\alpha\omega\left( e,t \right)>\text{thresh} \\ 0, &\text{otherwise} \end{aligned} \right.$ | ( 3 ) |
| --- | --- | --- |

Where $\alpha$ is a constant and $\omega\left( e,t \right)$ is a function tracking the cells recent firing rate to simulate neuronal adaptation (Kandel et al., 2000). Thus, an excitatory cell fires only if its membrane potential surpasses a predetermined threshold by a certain value determined by its recent activity. While excitatory cells fire in discrete spikes, inhibitory cells are modelled as graded response neurons to represent a cluster of local interneurons:

|  | $\phi\left( i,t \right)=\left\{ \begin{aligned} V\left( i,t \right), \text{if }V\left( i,t \right)>0 \\ 0, &\text{otherwise} \end{aligned} \right.$ | ( 4 ) |
| --- | --- | --- |

The amount of neural adaptation for *e*-cells $\omega\left( e,t \right)$ is defined as follows:

|  | $\tau_{ADAPT}\cdot\frac{d\omega\left( e,t \right)}{dt}=-\omega\left( e,t \right)+\alpha_{adapt}\phi\left( e,t \right)$ | ( 5 ) |
| --- | --- | --- |

Where $\tau_{ADAPT}$ is the time constant for adaptation. The solution $\omega\left( e,t \right)$ of Equation ( 5 ) is the low-pass-filtered output $\phi$ of cell $e$ and provides an estimate of the cell’s recent firing activity.

Activity in the network is regulated through local and global (area-specific) inhibition (Bibbig et al., 1995; Palm, 1982; Wennekers et al., 2006). This ensures that area and network activity remain within plausible physiological levels. Local inhibition is realized through *i*-cells, whereas global inhibition changes depending on excitatory spikes in an area, as defined by the following equation:

|  | $\tau_{GLOB}\cdot\frac{d\omega_{G}\left( e,t \right)}{dt}=-\omega_{G}\left( e,t \right)+\sum_{e\in\text{area}} \phi\left( e,t \right)$ | ( 6 ) |
| --- | --- | --- |

Where $\tau_{GLOB}$ is the time constant for global inhibition.

Synaptic connections between excitatory neurons either within or between linked areas are initialized with weak weights in the range of $\left[ 0,0.1 \right]$. They are limited to a local topographic neighborhood, with the probability of a synapse being created between two neurons decreasing with the distance between the cells according to a two-dimensional Gaussian function. The function is clipped to 0 outside the designated neighborhood size, which is a patch of 19x19 for excitatory and 5x5 for inhibitory neurons, thus creating a sparse and topographic connectivity typical for the mammalian cortex (Amir et al., 1993; Braitenberg & Schüz, 1998; Douglas & Martin, 2004) prior to training. The Gaussian function for within-area excitatory-excitatory connections has a center probability of 0.28, with $\sigma_{x}=\sigma_{y}=6.5$, the Gaussian function for between-area excitatory-excitatory connections has a center probability of 0.15 with $\sigma_{x}=\sigma_{y}=4.5$ and the one for inhibitory-excitatory connections has a center probability of 0.295, with with $\sigma_{x}=\sigma_{y}=2.0$.

This initial connectivity changes through a Hebbian learning mechanism which includes long-term potentiation (LTP) and long-term depression (LTD) (Artola et al., 1990). The model implementation includes both homo- and hetero-synaptic LTP and LTD. At each time step, the firing rate $\omega_{E}$ of cell $e$ is estimated as follows:

|  | $\tau_{Favg}\cdot\frac{d\omega_{E}\left( e,t \right)}{dt}=-\omega_{E}\left( e,t \right)+\phi\left( e,t \right)$ | ( 7 ) |
| --- | --- | --- |

Where $\tau_{Favg}$ is the time constant for firing rate estimation. The value of this firing rate determines whether or not a presynaptic cell is considered as active enough to induce LTP or homosynaptic LTD, following this equation:

|  | $\Delta w\left( i,j \right) \left\{ \begin{aligned} +\Delta, \text{if }\omega_{E}\left( i,t \right)\geq\theta_{pre}\text{ and} V\left( j,t \right)\geq\theta_{+}\left( \text{LTP} \right) \\ -\Delta, \text{if }\omega_{E}\left( i,t \right)\geq\theta_{pre}\text{ and} \theta_{-}\leq V\left( j,t \right)\geq\theta_{+}\left( \text{homosynaptic LTD} \right) \\ -\Delta, \text{if }\omega_{E}\left( i,t \right)<\theta_{pre}\text{ and} V\left( j,t \right)\geq\theta_{+} \left( \text{heterosynaptic LTD} \right) \\ 0 otherwise \end{aligned} \right.$ | ( 8 ) |
| --- | --- | --- |

Synaptic weights are thresholded to be within the range $[0.00000001 , 0.225]$.

**Table S1** Parameters used in simulation.

| Source | Description | Value |
| --- | --- | --- |
| Equations 1, 2 | Time constant (excitatory cells) Time constant (inhibitory cells) Total input rescaling factor Noise amplitude Global inhibition strength | $\tau=2.5 (time steps)$ $\tau=5 (time steps)$  $k_{1}=0.01$  $k_{2}=7\sqrt{\left( 24/\Delta t \right)}$  $k_{G}=0.8$ |
| Equation 3 | Spiking threshold | $\text{thresh}=0.18$ |
|  | Adaptation adjustment | $\alpha=800.$ |
| Equation 5 | Adaption time constant | $\tau_{ADAPT}=10\left( \text{time steps} \right)$ |
| Equation 5 | Adaption strength | $\alpha_{adapt}$=0.01 |
| Equation 6 | Global inhibition time constant | $\tau_{GLOB}=12\left( \text{time steps} \right)$ |
| Equation 7 | Rate-estimate time constant | $\tau_{Favg}=30\left( \text{time steps, training} \right)$  $\tau_{Favg}=5\left( \text{time steps, training} \right)$ |
| Equation 8 | Postsynaptic potential thresholds | $\theta_{+}=0.15\left( \text{LTP} \right)$  $\theta_{-}=0.14\left( \text{LTD} \right)$ |
|  | Presynaptic output activity required for any synaptic change: | $\theta_{pre}=0.05$ |
|  | Learning rate | $\Delta= 0.0008$ |
| Simulation environment | Time resolution of simulation | 0.5 |

## Appendix S2: Development of reverberation during extended training

We detected an upwards trend for reverberation of abstract concepts during Conceptual Learning. To test whether reverberation would continue to increase, we trained the models up to 10,000 trials. We took the reverberation across all extrasylvian areas at the end of the extended training period – 10,000 trials – to be the maximal achievable reverberation, and compared the reverberation measure every 500 trials to this maximum. For this analysis an additional model was removed (number 19, leading to 15 models in total), as most of its CAs merged by trial 3,000 in the *concrete* condition. Each comparison consisted in a two-sided t-test, leading to a total of 19 t-tests which were Bonferroni corrected within each semantic condition. Reverberation ceased increasing for concrete concepts at trial 7,500 (t(14)=2.72, p = 0.31, CI95=[0.0, 0.03]), and for abstract concepts at trial 1,000 (t(14)=3.42, p = 0.08, CI95=[0.15, 0.64]).

Figure S 1 CA reverberation for concrete (left) and abstract (right) concepts during extended Single Stage learning in the Conceptual Learning condition. The blue dots mark trials where there was no significant difference compared to the end of extended learning (10,000 trials), blue crosses mark trials with a significant difference.

## Appendix S3: Supplementary Results by Area

### Experiment 1 – Single stage learning

#### Reverberation

Figure S 2 CA reverberation throughout the Conceptual Learning condition in primary (*V1, *M1_L), secondary (*TO, *PM_L) and central (*AT, *PF_L) extrasylvian areas. B. CA reverberation throughout the Semantic Learning condition in primary, secondary and central extrasylvian areas.

During Conceptual Learning, we found a significant interaction between Semantic Type x Area x Training Trial for reverberation (F(198, 2,970) = 11, p < 0.01). Post-hoc t-tests showed a significant difference between concrete and abstract concepts for all Training Trials and Areas (all p < 0.001). The three-way interaction was also significant during Semantic Learning (F(198, 2,970) = 7.6, p < 0.001), however, the post-hoc t-tests stop showing a consistent significant difference in Primary areas between trials 60-180 and in trial 1,260, in Secondary areas between trials 60-140 and intermittently between trials 660-2,000, and in Central areas between trials 40-80 (see Figure S2 B; all other p < 0.05).

#### Structural changes

Figure S 3 Changes in CA composition throughout training in primary (*V1, *M1_L_), secondary (*TO, *PM_L_) and central (*AT, *PF_L_) extrasylvian areas. A. Number of unique (top), two-way shared (middle) and three-way shared (bottom) neurons during Conceptual Learning. B. Number of unique (top), two-way shared (middle) and three-way shared (bottom) neurons during Semantic Learning (with wordform). Concrete CAs are displayed in blue, abstract in red. The grey background highlights trials with non-significant differences between concepts.

During Conceptual Learning, we found a significant interaction between Semantic Type x Area x Training Trial for the number of unique (F(198, 2,970) = 39.9, p < 0.001), two-way shared (F(198, 2,970) = 39.2, p < 0.001) and three-way shared neurons (F(198, 2,970) = 16.8, p < 0.001). Post-hoc t-tests showed a significant difference between concrete and abstract concepts for all Training Trials and Areas for all neuron types (all p < 0.01) (see Figure S3 A).

During Semantic Learning, we found a significant interaction between Semantic Type x Area x Training Trial for the number of unique (F(198, 2,970) = 21.6, p < 0.001), two-way shared (F(198, 2,970) = 30.4, p < 0.001) and three-way shared (F(198, 2,970) = 70.6, p < 0.001) neurons. Post-hoc t-tests showed a significant difference between concrete and abstract concepts for all Training Trials and Area for all unique (except in Central areas at trials 1,260 and 1,920; all other p < 0.05) and two-way shared neurons (all p < 0.05) but crucially showed no significant difference for three-way shared neurons in Secondary areas from trial 260 onwards, and in Central areas from trial 100 onwards (see Figure S3 B).

### Experiment 2 – Two-stage learning

#### Reverberation

Figure S 4 Reverberation throughout both training stages in primary (*V1, *M1_L_), secondary (*TO, *PM_L_) and central (*AT, *PF_L_) extrasylvian areas.

The interaction between Semantic Type x Area x Training Trial for reverberation period was significant in Phase 1 (F(48, 1,104) = 3.02, p < 0.001) and Phase 2 (F(144, 3,312) = 18.01, p < 0.001). In Phase 1, the post-hoc t-tests showed no consecutive non-significant Trials. In Phase 2, results were significant until Trial 70 in Primary, and until Trial 60 in Secondary and Central areas. The difference becomes significant again at Trial 150 in Primary, and at Trial 100 in Secondary and Central areas (see Figure S4)

#### Structural changes

Figure S 5 Changes in CA composition throughout training in primary (*V1, *M1_L), secondary (*TO, *PM_L) and central (*AT, *PF_L) extrasylvian areas. Number of A. unique, B. two-way shared and C. three-way shared neurons during Conceptual Learning. Concrete CAs are displayed in blue, abstract in red. The grey background highlights trials with non-significant differences between concepts.

In Phase 1, we found a significant interaction between Semantic Type x Area x Training Trial for the number of unique (F(48, 1,104) = 9.68, p < 0.001), two-way shared (F(48, 1,104) = 8.8, p < 0.001) and three-way shared neurons (F(48, 1,104) = 14.52, p < 0.001). Post-hoc t-tests showed a significant difference between concrete and abstract concepts for all Training Trials and Areas for unique, two-way shared and three-way shared neurons (all p < 0.001) (see Figure S5).

In Phase 2, we found a significant interaction between Semantic Type x Area x Training Trial for the number of unique (F(144, 3,312) = 23.08, p < 0.001), two-way shared (F(144, 3,312) = 1.39, p=0.01) and three-way shared neurons (F(144, 3,312) = 91.22, p < 0.001).

In Phase 2, we were interested in the amount of training trials it took for significant differences between both concept types to disappear. Post-hoc t-tests showed no consecutive non-significant Trials when testing the difference between number of unique neurons in concrete and abstract concepts in Primary areas but ceased being significant at Trial 200 in Secondary and Central areas. For two-way shared neurons, the difference was significant for all Training Trials in Primary and Secondary areas, and until 200 in Central areas. For three-way shared neurons, the difference was significant for all Training Trials in Primary, until 150 in Secondary and until 100 in Central areas.

## References

Amir, Y., Harel, M., & Malach, R. (1993). Cortical hierarchy reflected in the organization of intrinsic connections in macaque monkey visual cortex. *The Journal of Comparative Neurology*, *334*(1), 19–46. https://doi.org/10.1002/cne.903340103

Artola, A., Bröcher, S., & Singer, W. (1990). Different voltage-dependent thresholds for inducing long-term depression and long-term potentiation in slices of rat visual cortex. *Nature*, *347*(6288), 69–72. https://doi.org/10.1038/347069a0

Bibbig, A., Wennekers, T., & Palm, G. (1995). A neural network model of the cortico-hippocampal interplay and the representation of contexts. *Behavioural Brain Research*, *66*(1-2), 169–175. https://doi.org/10.1016/0166-4328(94)00137-5

Braitenberg, V., & Schüz, A. (1998). *Cortex: statistics and geometry of neuronal connectivity*. Springer.

Douglas, R. J., & Martin, K. A. C. (2004). Neuronal circuits of the neocortex. *Annual Review of Neuroscience*, *27*, 419–451. https://doi.org/10.1146/annurev.neuro.27.070203.144152

Eggert, J., & van Hemmen, J. L. (2000). Unifying framework for neuronal assembly dynamics. *Physical Review. E, Statistical Physics, Plasmas, Fluids, and Related Interdisciplinary Topics*, *61*(2), 1855–1874. https://doi.org/10.1103/physreve.61.1855

Henningsen-Schomers, M. R., & Pulvermüller, F. (2022). Modelling concrete and abstract concepts using brain-constrained deep neural networks. *Psychological Research*, *86*(8), 2533–2559. https://doi.org/10.1007/s00426-021-01591-6

Kandel, E. R., Schwartz, J. H., & Jessel, T. M. (2000). *Principles of neural science.*

Palm, G. (1982). *Neural assemblies*. https://link.springer.com/content/pdf/10.1007/978-3-031-00311-0.pdf

Wennekers, T., Garagnani, M., & Pulvermüller, F. (2006). Language models based on Hebbian cell assemblies. *Journal of Physiology, Paris*, *100*(1-3), 16–30. https://doi.org/10.1016/j.jphysparis.2006.09.007

Wilson, H. R., & Cowan, J. D. (1972). Excitatory and Inhibitory Interactions in Localized Populations of Model Neurons. *Biophysical Journal*, *12*(1), 1–24. https://doi.org/10.1016/S0006-3495(72)86068-5

1. Adapted from Henningsen-Schomers and Pulvermüller (2022)
